# A comparison between different patient groups for diabetes management during phases of the COVID-19 pandemic: a retrospective cohort study in Ontario, Canada

**DOI:** 10.1186/s12875-024-02272-0

**Published:** 2024-01-27

**Authors:** A. Senthinathan, K. Tu, E. Stephenson, B. O’Neill, L. Lipscombe, C. Ji, D. A. Butt, J. Apajee, A. Train, N. Crampton

**Affiliations:** 1 Department of Family and Community Medicine, University of Toronto, Toronto, ON Canada; 2 North York General Hospital, Toronto, ON Canada; 3 Toronto Western Family Health Team, University Health Network, Toronto, ON Canada; 4 Department of Family and Community Medicine, St. Michael’s Hospital, Toronto, ON Canada; 5 MAP Centre for Urban Health Solutions, LiKa Shing Knowledge Institute, St. Michael’s Hospital, Toronto, ON Canada; 6 Women’s College Research Institute, Women’s College Hospital, Toronto, ON Canada; 7 Department of Medicine, University of Toronto, Toronto, ON Canada; 8 Scarborough Health Network, Toronto, ON Canada; 9 Department of Family Medicine, Queen’s University, Kingston, ON Canada

**Keywords:** Diabetes care, Type 2 diabetes, COVID-19, Virtual care

## Abstract

**Background:**

With the onset of the COVID-19 pandemic and the large uptake in virtual care in primary care in Canada, the care of patients with type 2 diabetes has been greatly affected. This includes decreased in-person visits, laboratory testing and in-person assessments such as blood pressure (BP). No studies have investigated if these changes persisted with pandemic progression, and it is unclear if shifts impacted patient groups uniformly. The purpose of this paper was to examine changes in diabetes care pre, early, and later pandemic across different patient groups.

**Methods:**

A repeated cross-sectional design with an open cohort was used to investigate diabetes care in adults with type 2 diabetes for a 6-month interval from March 14 to September 13 over three consecutive years: 2019 (pre-pandemic period), 2020 (early pandemic period), and 2021 (later pandemic period). Data for this study were abstracted from the University of Toronto Practice-Based Research Network (UTOPIAN) Data Safe Haven, a primary care electronic medical records database in Ontario, Canada. Changes in diabetes care, which included primary care total visits, in-person visits, hemoglobin A1c (HbA1c) testing, and BP measurements were evaluated across the phases of the pandemic. Difference in diabetes care across patient groups, including age, sex, income quintile, prior HbA1c levels, and prior BP levels, were assessed.

**Results:**

A total of 39,401 adults with type 2 diabetes were included in the study. Compared to the 6-month pre-pandemic period, having any in-person visits decreased significantly early pandemic (OR = 0.079 (0.076–0.082)), with a partial recovery later pandemic (OR = 0.162 (95% CI: 0.157–0.169). Compared to the pre-pandemic period, there was a significant decrease early pandemic for total visits (OR = 0.486 (95% CI: 0.470–0.503)), HbA1c testing (OR = 0.401 (95% CI: 0.389–0.413)), and BP measurement (OR = 0.121 (95% CI: 0.116–0.125)), with partial recovery later pandemic.

**Conclusions:**

All measures of diabetes care were substantially decreased early pandemic, with a partial recovery later pandemic across all patient groups. With the increase in virtual care due to the COVID-19 pandemic, diabetes care has been negatively impacted over 1-year after pandemic onset.

**Supplementary Information:**

The online version contains supplementary material available at 10.1186/s12875-024-02272-0.

## Introduction

In March 2020, the World Health Organization (WHO) declared the 2019 Coronavirus disease (COVID-19) a global pandemic [1]. With the onset of the COVID-19 pandemic, many health services, including care for patients with diabetes, were disrupted [2–6]. In Canada, family physicians are mainly responsible for the management of individuals with type 2 diabetes, which is consistently one of the most common reasons for a primary care visit [2, 7]. Both patients and healthcare providers were tasked with balancing the risks and benefits associated with in-person visits [3, 6]. A previous study conducted in Ontario assessed changes in primary care visits during the COVID-19 pandemic and found a large shift from in-person to virtual visits with over three quarters of primary care visits being virtual and over 70% of visits billed for diabetes care were conducted virtually [2]. Although prior to the pandemic, virtual visits were not normally offered in primary care, with the onset of the pandemic virtual appointments were encouraged for patients with diabetes as they are at increased risk of COVID-19 infections and severe complications requiring hospitalization [4, 8, 9]. As such, many primary care providers adopted a “virtual first” approach offering appointments via telephone or video before deciding to see a patient in-person [2, 6, 9]. Other strategies included extending the time between follow-up appointments for patients whose disease was well controlled [3, 5, 6].

However, many aspects of diabetes management and care cannot be conducted virtually and require in-person physical examinations [5, 7]. A study conducted in Ontario, Canada found that during the first six months of the pandemic, adults with type 2 diabetes had a significant reduction in the number of in-person visits and a significant increase in the number of virtual visits with their family physician [5]. It also found during this period a decrease in many key components of diabetes management including eye exams, hemoglobin A1c (HbA1c) tests, and low-density lipoprotein (LDL) cholesterol tests [5]. Hence, virtual care visits may not meet the needs of individuals with diabetes, especially those with inadequate glycemic control who are at-risk of diabetes complications and require frequent assessments and in-person visits [4, 8].

Although there were shifts in type 2 diabetes care early in the pandemic, what remains unclear is whether these shifts persisted as the pandemic progressed and if it occurred uniformly across different patient groups. Given recommendations for certain patient groups, such as elderly patients, those with inadequate HbA1c levels or elevated blood pressure (BP), receive more frequent visits, BP measurements and HbA1c assessments [7, 10–13], it is possible some patient groups were more affected, while others were less affected. We hypothesize that measures of diabetes care compared to pre-pandemic levels decreased during the early pandemic and continued to be depressed during the later pandemic, with elderly individuals, individuals with inadequate HbA1c levels, or individuals with elevated BP experiencing greater shifts for diabetes care during the pandemic. The purpose of this paper is to examine shifts in type 2 diabetes care during different phases of the pandemic across different patient groups (age, sex, HbA1c levels, and BP measurement).

## Methods

### Study design

We used a repeated cross-sectional design with an open cohort of patients with type 2 diabetes to investigate trends in diabetes care pre-pandemic (March 14-September 13, 2019), early pandemic (March 14- September 13, 2020), and later pandemic (March 14- September 13, 2021). We investigated shifts in type 2 diabetes care, which included in-person visits, all visits, BP measures, and HbA1c, across different patient groups. Patient characteristics assessed to create patient groups included age, sex, neighbourhood income quintile, previous HbA1c levels (in the preceding 6-months), and previous BP control (in the preceding 6-months). Data from an electronic medical record (EMR) database from a fixed cohort of family physicians (*n* = 280) were extracted up to mid-September 2021 to identify patients with type 2 diabetes and measures of diabetes care.

### Setting

Data for this study were from the University of Toronto Practice-Based Research Network (UTOPIAN) Data Safe Haven, a primary care EMR database that includes records from family medicine clinics in Ontario, Canada [14]. In the UTOPIAN database, over 70% of family practices are in the Greater Toronto Area [14]. UTOPIAN patients are slightly more female, elderly and higher income quintile than the general Ontario population [2] but this is thought to be typical of the type of patients that see family physicians in general and not something specific to UTOPIAN patients.

### Participants

We identified a cohort of adult family practice patients (18 years or older) with type 2 diabetes who met minimum standards for data quality and completeness [14], who were actively receiving care from a family physician contributing data to UTOPIAN (defined as at least 2 visits within the past 3 years), and who were deemed to have type 2 diabetes based on documentation in the cumulative patient profile, laboratory testing history, medication history, and billing records (See Appendix A). The case definition for type 2 diabetes was based on previously validated algorithms for primary care EMR data [15–17].

### Outcome measures

Measures of diabetes care (total visits, in-person visits, HbA1c testing, and BP assessments) and disease control (HbA1c and BP levels) were assessed in the same time period from March 14 to September 13 over three consecutive years: in 2019 (pre-pandemic period), 2020 (early pandemic period), and 2021 (late pandemic period). An observation period of 6 months was used because guidelines for management of diabetes recommend assessments every 3–6 months [7, 11].

#### Primary care visit

As in other studies using the UTOPIAN database [2, 14, 18], billing codes were used to define the occurrence of a family practice visit and to classify visits based on format of care delivery (in-person, virtual) (See Appendix B).

#### Blood Glucose levels

HbA1c tests captured in the EMR were used to assess blood glucose levels. The proportion of patients tested at least once within a 6-month period was used as a measure of quality of care. Patients with a recent hemoglobin A1c test result were further categorized based on whether their most recent result indicated optimal (< 7.0%), suboptimal (≥ 7.0%- <8.5%), or inadequate (≥ 8.5%) glucose control [7].

#### Blood pressure

Systolic (sBP) and diastolic blood pressure (dBP) readings in the EMR were used to determine the proportion of patients with at least one BP assessment within a 6-month period and their BP control (elevated BP defined as sBP ≥ 130 mmHg or dPB ≥ 80 mmHg, normal BP defined as sBP < 130 and dBP < 80) based on the most recent assessment [7].

### Patient characteristics

Patient characteristics such as sex and age were extracted directly from the EMR. Postal codes were used to determine neighborhood income quintiles based on the Statistics Canada Postal Code Conversion File [19]. For each time period (pre-pandemic, early pandemic, and later pandemic), in the preceding six months the most recent HbA1c test result was used to determine pre-existing glucose control, and most recent BP recorded was used to determine pre-existing BP control (Appendix C). It should be noted that pre-existing glucose control and pre-existing BP control were used to stratify the cohort to assess differences between those with varying levels of control, as well whether a HbA1c test and BP assessment were conducted during each 6-month time period were listed as outcome measures for diabetes care in this study.

### Statistical analysis

For each time period (pre-pandemic, early pandemic, and later pandemic), we estimated the proportion of patients who had at least one family physician visit of any format, at least one family physician visit in-person, at least one HbA1c test result, and at least one BP measurement. Among those with at least one HbA1c test result, we estimated the proportion of results that were in the optimal, suboptimal, and inadequate control ranges. Among those with at least one BP measurement, we estimated the proportion of results that were in elevated and normal control ranges. We used logistic generalized estimating equation regression models with an exchangeable correlation structure to determine the patient characteristics associated with diabetes care. Multiplicative interaction terms (e.g., sex × time period) were added to the regression models to test whether the effects of patient characteristics on measures of diabetes care changed during the pandemic, relative to pre-pandemic. Models were adjusted for age, sex, income, pre-existing glucose control and pre-existing BP control. Analyses were performed in SAS version 9.4.

## Results

### Sample characteristics

Records from 39,401 patients with type 2 diabetes were included in this study. Patient demographics were consistent across the pre-pandemic and both pandemic periods (Table 1).

**Table 1:** Patient characteristics

		Pre-pandemic (*N* = 25,361)		Early Pandemic (*N* = 27,463)		Later Pandemic (*N* = 28,331)	
Variable		**n**	**%**	**n**	**%**	**n**	**%**
Age group							
	18–49 years	3,469	**13.7**	3,666	**13.3**	3,666	**12.9**
	50–64 years	8,924	**35.2**	9,329	**34.0**	9,350	**33.0**
	65–79 years	9,304	**36.7**	10,239	**37.3**	10,709	**37.8**
	80 years and older	3,664	**14.4**	4,229	**15.4**	4,606	**16.3**
Sex	Female	12,394	**48.9**	13,502	**49.2**	14,017	**49.5**
	Male	12,967	**51.1**	13,961	**50.8**	14,314	**50.5**
Neighborhood income quintile	Lowest income	7,139	**28.1**	7,712	**28.1**	7,900	**27.9**
	Moderately low income	5,197	**20.5**	5,630	**20.5**	5,765	**20.3**
	Middle income	4,127	**16.3**	4,476	**16.3**	4,637	**16.4**
	Moderately high income	3,823	**15.1**	4,195	**15.3**	4,398	**15.5**
	Highest income	4,390	**17.3**	4,705	**17.1**	4,851	**17.1**
	Missing	685	**2.7**	745	**2.7**	780	**2.8**

### Pandemic effects on visit rates and care delivery format

**Fig. 1:**
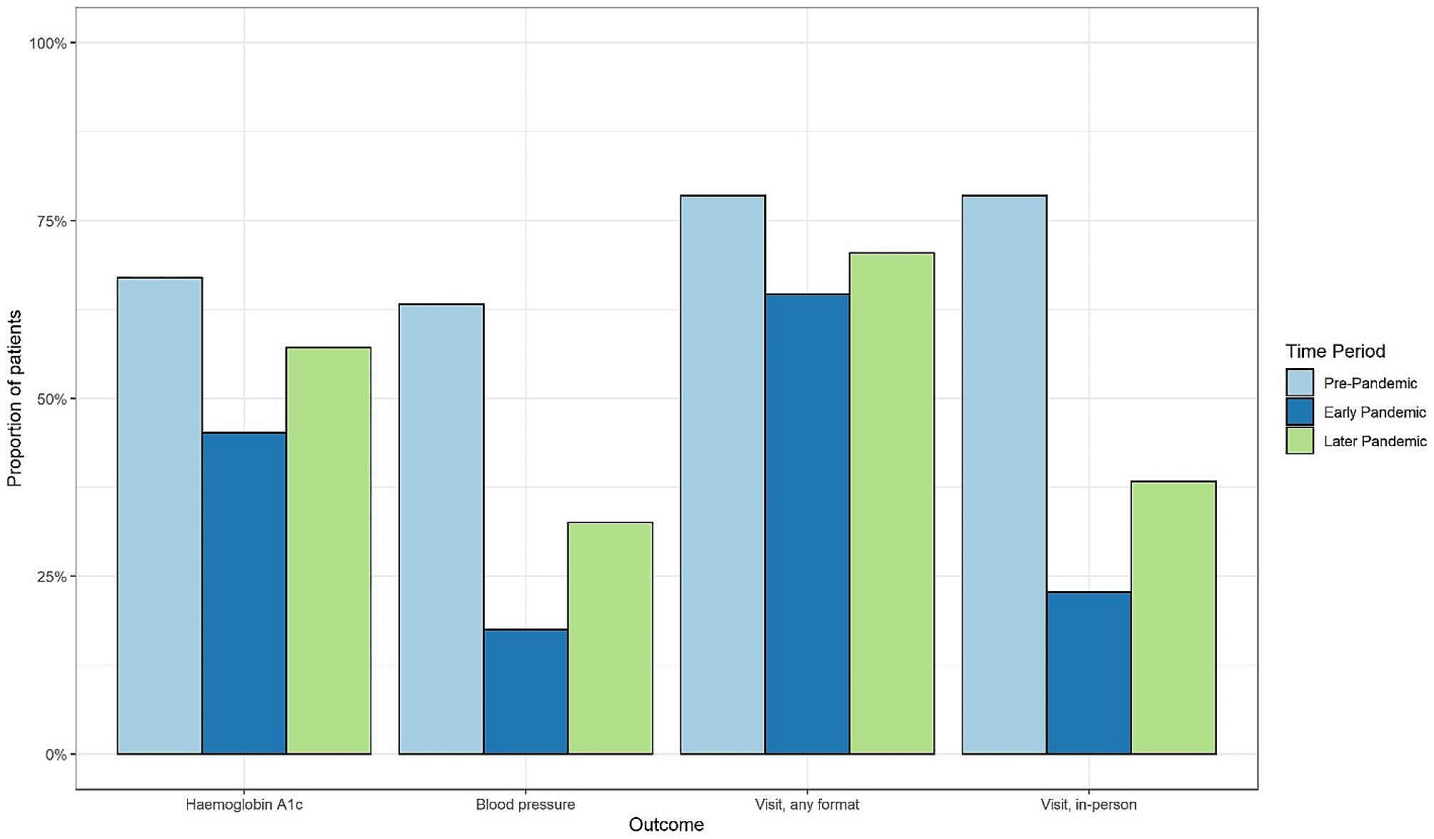
Proportion of patients with HbA1c testing, BP assessments, in-person visits and visits during pre-pandemic, early pandemic and later pandemic

The number of patients visiting their family physician in-person dropped substantially following the onset of the pandemic (Table 2; Fig. 1). Within the assessed 6-month time period, in the early pandemic period, 22.7% of patients had at least one in-person visit compared to 78.5% pre-pandemic (OR = 0.08 (95% CI: 0.08–0.08), *p* < 0.0001) (Table 2). However, 64.6% of patients had at least one virtual or in-person visit in the early pandemic period. In the later pandemic period 38.3% of patients had at least one in-person visit, and 70.5% had had at least one virtual or in-person visit.

**Table 2:** Change in patients receiving diabetes care during the early and later pandemic compared to pre-pandemic

		Pre-pandemic		Early Pandemic		Late Pandemic		Odd Ratio (OR) (95% CI), *p*-value	Odd Ratio (OR) (95% CI), *p*-value
		(*N* = 25,361)		(*N* = 27,463)		(*N* = 28,331)		Pre-Pandemic vs. Early Pandemic	Pre-Pandemic vs. later Pandemic
**Measure**		n	**%**	n	**%**	n	**%**		
**Any visit**									
	None	5,454	**21.5**	9,712	**35.4**	8,362	**29.5**	**0.49** (0.47–0.50), < 0.0001	**0.60** (0.58–0.62), < 0.0001
	At least one	19,907	**78.5**	17,751	**64.6**	19,969	**70.5**		
**In-person visit**									
	None	5,454	**21.5**	21,216	**77.3**	17,475	**61.7**	**0.08** (0.08–0.08), < 0.0001	**0.16** (0.16–0.17), < 0.0001
	At least one	19,907	**78.5**	6,247	**22.7**	10,856	**38.3**		
**HbA1c test**									
	None	8,389	**33.1**	15,063	**54.8**	12,137	**42.8**	**0.40** (0.39–0.41), < 0.0001	**0.63** (0.61–0.65), < 0.0001
	At least one	16,972	**66.9**	12,400	**45.2**	16,194	**57.2**		
**Blood pressure**									
	None	9,329	**36.8**	22,663	**82.5**	19,105	**67.4**	**0.12** (0.17 − 0.13), < 0.0001	**0.27** (0.26–0.28), < 0.0001
	At least one	16,032	**63.2**	4,800	**17.5**	9,226	**32.6**		

### Pandemic effects on HbA1c testing and BP assessments

During the 6-month pre-pandemic period, 66.9% of patients had their HbA1c levels tested and 63.2% of patients had their BP documented at least once (Table 2; Fig. 1). Early pandemic, these levels dropped to 45.2% (OR = 0.401 (95% CI: 0.39–0.41), *p* < 0.0001), and 17.5%, respectively (OR = 0.121(95% CI: 0.17 − 0.13), *p* < 0.0001) (Table 2). Later pandemic, testing improved to 57.2% for HbA1c tests (OR = 0.63 (95% CI: 0.61–0.65), *p* < 0.0001) and 32.6% for BP assessments (OR = 0.27 (95% CI: 0.26–0.28), *p* < 0.0001); however, these were still lower than pre-pandemic levels (Table 2). Although fewer patients were tested, among those tested, the proportion of individuals in the glucose and BP control categories remained relatively stable over time (Table 3).

**Table 3:** Change in levels of disease control among those assessed in the pre-pandemic, early pandemic and later pandemic

		Pre-pandemic			Early Pandemic				Late Pandemic		
Variable	Level	N	**% of all patients**	***% of those tested***	N		**% of all patients**	***% of those tested***	N	**% of all patients**	***% of those tested***
Prior HbA1c test	Optimal (< 7.0%)	9,346	**36.9**	***55.1***		7,218	**26.3**	***58.2***	9003	**31.8**	***55.6***
	Suboptimal (≥ 7.0% and < 8.5%)	5,713	**22.5**	***33.7***	3,911		**14.2**	***31.5***	5361	**18.9**	***33.1***
	Inadequate (≥ 8.5%)	1,913	**7.5**	***11.3***	1,271		**4.6**	***10.3***	1830	**6.5**	***11.3***
	Not tested	8,389	**33.1**	***-***	15,063		**54.8**	***-***	12,137	**42.8**	***-***
Prior BP assessment	Elevated (sBP > = 130 or dBP > = 80)	9125	**36.0**	***56.9***	2921		**10.6**	***60.9***	5466	**19.3**	***59.2***
	Normal (sBP < 130 and dBP < 80)	6907	**27.2**	***43.1***	1879		**6.8**	***39.1***	3760	**13.3**	***40.8***
	Not assessed	9329	**36.8**	**-**	22,663		**82.5**	**-**	19,105	**67.4**	**-**

### Diabetes care shifts across patient groups during the pandemic

**Fig. 2:**
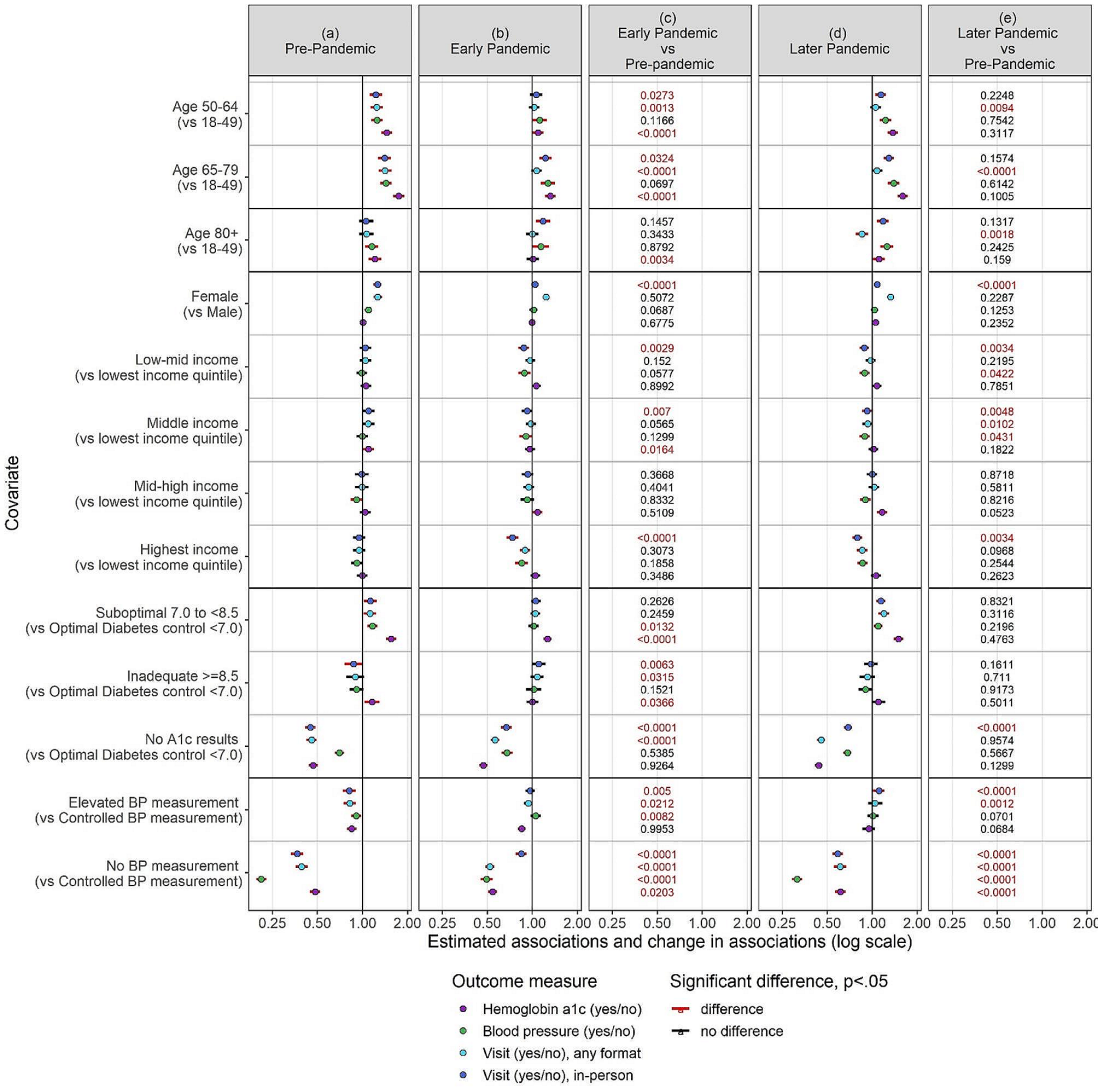
Odds Ratio comparing pre-pandemic to early pandemic and later pandemic across different patient groups

**Fig. 3:**
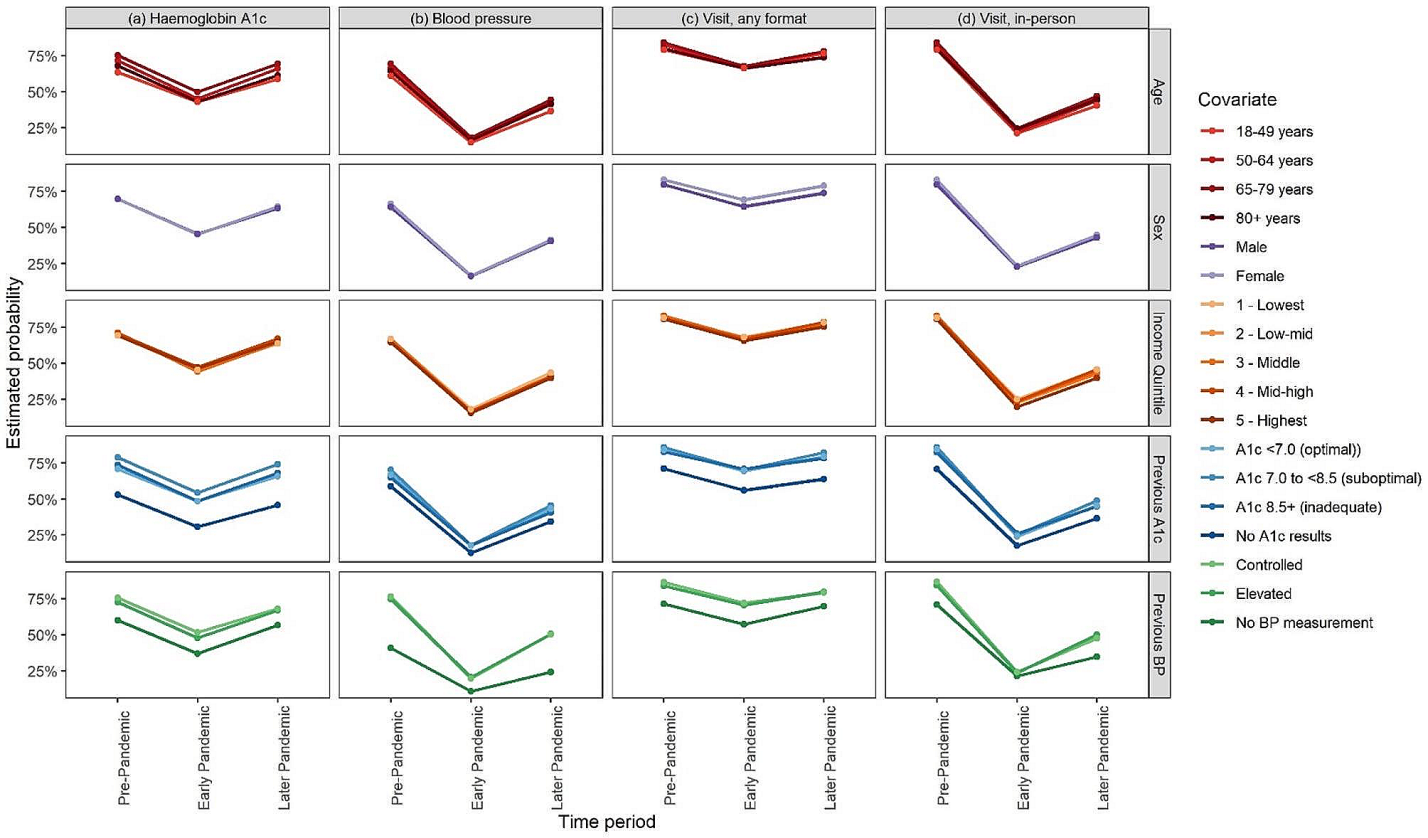
Diabetes care pre -pandemic, early pandemic and later pandemic, by age, sex, income, and prior HbA1c levels, and prior BP control

All patient groups for sex, age, income quintile, glucose control, and BP control experienced a decrease in any visits, in-person visits, HbA1c testing and BP assessments early pandemic, compared to pre-pandemic levels, with a partial recovery later pandemic (Table 4; Fig. 2, and Fig. 3). Prior to the pandemic, patients who were female, and older were more likely to visit their family physician and have their BP assessed; patients who were older were also more likely to have their HbA1c levels checked (Fig. 2a). These factors continued to be associated with diabetes care during the pandemic (Fig. 2b and d), but the strength of these associations changed (Fig. 2c and e). Early and later pandemic patients who had in-person visits were more likely to be male (*p* < 0.001), and older (*p* < 0.05 for patients age 50–64 and age 65–79 compared to patients age 18–49 in 2020), than patients who were visiting in-person before the pandemic (Fig. 2c and e).

**Table 4:** Change in diabetes care measures across different patient groups pre-pandemic, early pandemic and later pandemic

		HbA1c Testing			BP Assessment			Any visit				In-person visit			
Variable		Pre-pandemic	Early Pandemic	Later pandemic	Pre-pandemic	Early Pandemic	Later pandemic	Pre-pandemic	Early Pandemic	Later pandemic	Pre-pandemic		Early Pandemic	Later pandemic	
**Age group**	18–49 years	63.6%	43.0%	58.8%	61.1%	14.8%	36.4%	79.0%	66.4%	76.8%	79.2%		21.1%	40.5%	
	50–64 years	71.7%	45.2%	66.2%	66.3%	16.3%	41.2%	82.4%	67.0%	77.7%	82.4%		22.1%	43.7%	
	65–79 years	75.3%	49.9%	69.5%	69.3%	18.0%	44.3%	84.2%	67.8%	78.0%	84.3%		24.6%	46.7%	
	80+	67.9%	43.2%	61.2%	64.4%	16.5%	41.7%	80.1%	66.4%	73.8%	80.1%		24.0%	44.5%	
**Sex**	Female	69.9%	45.3%	64.6%	66.4%	16.5%	41.3%	83.2%	69.2%	79.1%	83.3%		23.3%	44.8%	
	Male	69.7%	45.4%	63.4%	64.3%	16.3%	40.4%	79.7%	64.5%	74.0%	79.8%		22.6%	43.0%	
**Neighborhood income quintile**	Lowest income	69.5%	45.3%	63.7%	66.8%	17.9%	43.4%	81.6%	68.2%	78.1%	81.7%		24.9%	45.5%	
	Moderately low income	70.6%	46.8%	65.3%	66.6%	16.1%	40.5%	82.3%	67.5%	77.7%	82.3%		22.5%	42.5%	
	Middle income	71.4%	44.3%	64.2%	66.7%	16.4%	40.5%	82.9%	67.7%	76.9%	83.0%		23.4%	43.5%	
	Moderately high income	70.3%	47.2%	67.2%	64.7%	16.7%	40.8%	81.5%	66.9%	78.6%	81.5%		23.6%	45.5%	
	Highest income	69.4%	46.4%	65.0%	64.9%	15.5%	39.8%	80.8%	65.7%	75.4%	80.9%		19.6%	39.9%	
	Missing	67.6%	42.0%	58.3%	62.2%	15.6%	40.2%	79.8%	65.3%	72.7%	80.1%		23.6%	46.3%	
**Prior HbA1c test**	Optimal < 7.0	70.7%	48.6%	65.9%	67.0%	17.6%	43.3%	84.3%	69.4%	79.5%	84.5%		24.1%	45.4%	
	Suboptimal 7.0 to < 8.5	79.0%	54.4%	74.3%	70.3%	17.8%	45.5%	85.7%	70.3%	82.3%	86.0%		25.0%	48.8%	
	Inadequate > = 8.5	73.7%	48.6%	68.0%	64.9%	17.9%	40.8%	82.8%	70.9%	78.3%	82.6%		25.9%	45.0%	
	Not tested	53.0%	30.8%	45.8%	58.7%	12.6%	34.2%	71.0%	56.0%	63.9%	70.9%		17.5%	36.5%	
**Prior BP assessment**	Normal (sBP < 130 and dBP < 80)	75.7%	51.8%	68.1%	76.6%	19.6%	50.3%	86.6%	71.9%	79.2%	86.9%		24.1%	47.4%	
	Elevated (sBP > = 130 or dBP > = 80)	72.5%	47.7%	66.9%	74.9%	20.4%	50.6%	84.1%	70.7%	79.9%	84.4%		23.5%	49.9%	
	Not assessed	60.0%	36.8%	56.6%	40.7%	10.7%	24.1%	71.6%	57.1%	69.9%	70.8%		21.1%	34.6%	

Early pandemic, prior HbA1c levels were no longer associated with having at least one visit and at least one BP measurement documented (Fig. 2b). Early pandemic, patients with optimal and suboptimal HbA1c levels were equally likely to have a visit with their family physician (OR = 1.05 (95% CI: 0.98–1.12)) and have their BP measured (OR = 1.02 (95% CI: 0.94–1.10)) (Fig. 3). Later pandemic, the predictive effect of prior HbA1c levels was the same as pre-pandemic (Fig. 2d). Pre-pandemic, those with elevated BP compared with controlled BP, were less likely to have an in-person visit (OR = 0.74 (95% CI: 0.64–0.83), *p* < 0.0001), and any visit (OR = 0.79 (95% CI: 0.68–0.91), *p* = 0.001) (Fig. 2a), however early pandemic those with elevated BP and controlled BP were equally as likely to have any visits or in-person visits.

## Discussion

The COVID-19 pandemic led to considerable disruption to the provision of standard clinical care, including primary care for individuals with type 2 diabetes. Our study aimed to analyze type 2 diabetes care as it shifted throughout the pandemic across different patient groups. This study found family physicians rapidly increased their use of virtual appointments to maintain care for their patients with diabetes. With the pandemic’s onset, for the 6-month time period early pandemic we found a significant drop for in-person visits to 22.7% from 78.5% pre-pandemic 6-month time period, however the increase in virtual visits may have mitigated as there was only a drop from 78.5 to 64.7% for total visits early pandemic from pre-pandemic levels. The later pandemic 6-month time period showed a slight recovery in both in-person and total visit levels. While the proportion of patients with optimal glucose control and blood pressure tested during the early pandemic was unchanged from pre-pandemic levels, the number of patients tested had decreased substantially with only a partial recovery later pandemic. A decrease in diabetes care measures early pandemic with a rebound later pandemic was observed in all patient groups.

The findings from our study are consistent with other studies evaluating changes in diabetes management in primary care during the pandemic in Ontario, Canada [4, 5, 20]. A study conducted in Ontario, found for adults with type 2 diabetes in the first six-months of the pandemic a significant reduction in the number of in-person primary care visits (RR = 0.53 (95% CI: 0.53–0.53), *p* < 0.0001), an increase in virtual visits (RR = 36.42 (95% CI: 35.95–36.89), *p* < 0.0001), and a decrease in HbA1c testing (RR = 0.72 (95% CI: 0.71–0.72), *p* < 0.0001) [5] compared to pre-pandemic levels (March 2019 to September 2019), which is in line with findings from our study, with our study also demonstrating a sustained reduction. Another study conducted in Ontario investigating foot complications, amputations, and other measures of care in individuals with diabetes, found significant decreases during the onset of the COVID-19 pandemic in rates of comprehensive in-person diabetes care assessments, including HbA1c testing compared to 2019–2020 levels [20]. For the rate of HbA1c testing, the study also found the initial drop to 41% during the first two months of the pandemic recovered to 84% of the 2019 baseline level between July and October 2020 [20], which is similar to the findings from our study which demonstrated a drop early pandemic from pre-pandemic levels, with a recovery in the later pandemic period. Comprehensive in-person diabetes care assessments also recovered slightly from 28 to 50% of 2019 baselines levels between July and October 2020 [20]. The findings from this study are similar to the findings from our study which also showed a decrease in HbA1C measurements and diabetes care during the onset of COVID-19 with a partial recovery later in the pandemic [20].

Although our study showed similar changes early pandemic in diabetes care to other literature in Ontario, it also showed a partial recovery in diabetic care measures as the pandemic progressed. To our knowledge, there are no other published studies that investigates diabetes care beyond the first year of the COVID-19 pandemic in Ontario, or anywhere else in North America. Our study highlights that in the later pandemic phase, a year after the pandemic onset, the initial gap in diabetes care created early pandemic began to close, as number of visits, in-person visits, HbA1c testing, and BP assessments improved. This study also included an additional measure of diabetes care of BP assessments not previously investigated in other studies. The Canadian Diabetes Clinical Practice Guidelines outline the importance of conducting BP assessments in individuals with diabetes, hence the investigation of changes to BP assessments during the pandemic is important [7]. Furthermore, no other studies have assessed the changes of diabetes care across different patient characteristics. Understanding the impact of how patient characteristics may have resulted in differential experiences due to the COVID-19 pandemic provides insight into if certain groups of patients were more negatively impacted and might therefore require further interventions to improve their care. Although our study found there were some differences across patient characteristic groups with diabetes care during the different phases of the pandemic, all patient groups experienced similar changes with decreased total visits, in-person visits, HbA1c testing, and BP assessments early pandemic, with a partial recovery later pandemic. This suggests no one group experienced substantially greater impacts to diabetes care due to the pandemic, which is reassuring as it confirms pre-existing differences in diabetes care were not exacerbated. In fact, those with inadequate HbA1c levels and elevated BP, compared to those with optimal HbA1c and controlled BP respectively, demonstrated a smaller decrease in diabetes care during the early pandemic. This could be due to primary care physicians prioritizing those with the greatest needs, or due to patients with the greatest needs actively seeking care during the pandemic.

Due to the rapid adoption of virtual care and related technologies, the pandemic may have paved an avenue for remote testing for diabetes management and care [21–24]. Our study found that as in-person visits increased in the late pandemic period, HbA1c and BP testing rates also improved compared to early pandemic. However, it is possible that BP be monitored remotely using at home BP monitors, and blood glucose level also be measured remotely using continuous glucose monitors or other at home blood glucose monitors [21–24]. With the rapid adoption of remote monitoring for diabetes care, this may have reduced the need for patients to seek primary care in-person. As such, in-person visits for diabetes management may not return to pre-pandemic levels, and virtual visits will continue. Although the pandemic may have initial negatively impacted diabetes care and management, it may also be the catalyst for the integration of virtual care and remote monitoring for diabetes care and management, possibly resulting in improved patient outcomes.

### Limitations

Our study used a convenience sample of primary care providers in Ontario, as only patients with visits to family physician clinics that contribute to UTOPIAN were included. Patients in UTOPIAN practices are primarily from academic family health teams and include fewer family physicians with independent community practices [25]. As such, findings from this study may not be generalizable to other primary care populations in Ontario. Additionally, patients in this study may have sought medical care outside of their family physicians from other healthcare providers, such as specialists or walk-in clinics, which would not be captured in our study. As such, diabetes care measures captured in this study may not be reflective of the actual care individuals with diabetes received during the pandemic. Furthermore, this was a retrospective study, as such variables obtained for BP assessments were identified only if entered in structured fields. If BP assessments were written in the unstructured progress notes rather than the designated structured fields for these values, they would have been missed.

## Conclusion

Type 2 diabetes care is commonly managed by primary care clinicians, and with the onset of the COVID-19 pandemic, care was redirected to virtual appointments. Our study showed early pandemic in-person visits dropped by 55.8%, from 78.5 to 22.7%. We also demonstrated later pandemic, early pandemic, patients with type 2 diabetes had an absolute reduction by 9.7% in HbA1c testing, and by 30.6% in BP assessments, from pre-pandemic levels. Encouragingly, we found during the later pandemic, measures of diabetes care recovered, perhaps as the healthcare system began to adapt to the changes resulting from the COVID-19 pandemic. Our study showed similar changes across different patient groups, including patients with elevated BP and inadequate HbA1c suggesting primary care providers may aim to prioritize high-risk groups during times of low resource availability. Future studies should continue to investigate these shifting dynamics impact on primary care, including exploring short-term and long-term effects, to inform policymakers and healthcare providers on how to best optimize primary care for individuals with diabetes.

### Electronic supplementary material

Below is the link to the electronic supplementary material.

Supplementary Material 1

## Data Availability

The research ethics approval for the use of UTOPIAN data does not permit making the data publicly available. Researchers interested in accessing EMR data from the UTOPIAN Data Safe Haven for research can apply to do so at: https://www.dfcm.utoronto.ca/getting-utopian-support.
