# Supplementary material for: A comparison between different patient groups for diabetes management during phases of the COVID-19 pandemic: a retrospective cohort study in Ontario, Canada
Source: BMC Prim Care. 2024 Jan 27;25:43. doi: 10.1186/s12875-024-02272-0 (PMC10821561; doi:10.1186/s12875-024-02272-0)
Supplement: Supplementary file 1 — Supplementary Material 1 [file 12875_2024_2272_MOESM1_ESM.docx]

**Supplementary Materials**

**Appendix A: Identifying patients with type 2 diabetes**

Patients with diabetes (type 1 and 2) are identified based on the following criteria. The date case detected is the earliest date when at least one of these criteria is met.

1. Free text documentation of diabetes in the past or present health condition section of the cumulative patient including the following terms:

| Include | Do Not Include |
| --- | --- |
| Diabetes | Insipid |
| Niddm | Chemical induced |
| Dm/Dm2/Dmii | Pregnancy |
| T2dm | Maternal |
| Iddm | Gestation |
| T1d/t1dm | Borderline |
| T2d/dka/type2dm | Pre-diabetes |
|  | Prednisone |

**OR**

1. Two consecutive eligible labs of the same type that are less than two years apart.
   1. Eligible labs:
      1. HbA1c >= 6.5%
      2. FBS >= 7.0 mmol/L
      3. RBS >= 11.1 mmol/L
   2. Exclude labs during **pregnancy period^1^**

**OR**

1. Diabetes medication^2^ (oral hypoglycemic or insulin) prescribed at any time in the EMR

a. Exclude metformin and insulin during **pregnancy period^1^**

b. Exclude metformin for female patients who are 50 years old or younger

Rational: Metformin may be prescribed for polycystic ovarian syndrome, hence in the medication list metformin for female patients who are 50 years old or younger was not included as criteria for identifying diabetics, female patients under 50 could have been classified as diabetic if they met HbA1c or glucose criteria or by other medications but metformin alone in these patients was insufficient to classify females under 50 as diabetic.

1. OHIP billing codes specifically for management of patients with diabetes at any point in the EMR
   1. Q040 Diabetes Management Incentive (DMI)
   2. K030 Diabetic Management Assessment (DMA)
2. Manual review of the CPP text in patient charts was conducted to exclude cases of type 1 diabetes to create a final cohort of cases of type 2 diabetes.

**Notes:**

**^1^ Pregnancy period** (Note: patients may develop gestational diabetes which may resolve after delivery. Therefore we do not include diabetes lab tests readings taken during the time of pregnancy)**:**

1. Start time (the earliest of):
   1. The time when a female patient’s ß-hCG lab test >=5.0. (If more than one ß-hCG in a 100 day time period use the date of the earliest one)

**OR**

- 1. 30 weeks before a female patient‘s pregnancy related oral glucose tolerance test

1. End time (the latest of):
   1. 40 weeks after a female patient’s ß-hCG lab test >= 5.0

**OR**

- 1. 20 weeks after a female patient‘s pregnancy related oral glucose tolerance test lab test

**^2^ Diabetes medications:**

| Class | Generic | Brand Name |
| --- | --- | --- |
| Biguanide | metformin | Glucophage, Glumetza, Glycon, Riomet |
| Incretin (Dipeptidyl Peptidase-4 inhibitors) (DPP-4) | alogliptin | Nesina |
|  | linagliptin | Trajenta, Tradjenta |
|  | saxagliptin | Onglyza |
|  | sitagliptin | Januvia, Ristaben, Tesavel, Xelevia |
| Incretin (Glucagon-like Peptide-1 receptor agonists) (GLP-1) | albiglutide | Eperzan, Tanzeum |
|  | dulaglutide | Trulicity |
|  | exenatide | Bydureon, Bydureon Bcise, Byetta |
|  | liraglutide | Saxenda, Victoza |
|  | lixisenatide | Adlyxine, Adlyxin, Lyxumia |
|  | semaglutide | Ozempic, Rybelsus |
| SGLT-2 Inhibitors | canagliflozin | Invokana, Invokamet |
|  | dapagliflozin | Forxiga, Edistride, Farxiga |
|  | empagliflozin | Jardiance |
|  | ertugliflozin | Steglatro |
| Alpha-glucosidase Inhibitor | acarbose | Glucobay, Mar-Acarbose, Precose |
| Insulin | insulin | Sulphated Insulin, Entuzity, Humulin, Iletin, Novolin, Novolinset, Velosulin, Hypurin, Lente Insulin, NPH Insulin, Regular Insulin |
|  | insulin aspart | Fiasp, Novomix, Novorapid, Novolog |
|  | insulin degludec | Tresiba |
|  | insulin detemir | Levemir |
|  | insulin glargine | Basaglar, Lantus, Toujeo Abasaglar, Semglee |
|  | insulin glulisine | Apidra |
|  | insulin lispro | Admelog, Humalog, Liprolog, Lyumjev |
| Insulin secretagogue (Sulfonylureas) | acetohexamide | Dimelor |
|  | chlorpropamide | Diabinese, Novo-Propamide |
|  | glibenclamide | Glyburide, Diabeta, Euglucon, Med Glybe, Mylan-Glybe, Glynase, Miconase |
|  | gliclazide | Diamicron, Diamicron Mr, Glic, Amaryl, Glimepiride |
|  | tolbutamide | Mobenol, Novo-Butamide, Orinase |
| Insulin secretagogue (Meglitinides) | nateglinide | Starlix |
|  | repaglinide | Gluconorm, Prandin |
|  |  |  |
|  |  |  |
| Class | **Generic** | **Brand Name** |
| Thiazolidinediones | pioglitazone | Actos, Glustin |
|  | repaglinide | Gluconorm, Novonorm |
|  | rosiglitazone | Avandia |
| CombinationAgents | metformin and ertugliflozin | Segluromet |
|  | ertugliflozin and sitagliptin | Steglujan |
|  | glimepiride and rosiglitazone | Avandaryl |
|  | insulin degludec and liraglutide | Xultophy |
|  | insulin glargine and lixisenatide | Soliqua |
|  | linagliptin and empagliflozin | Glyxambi |
|  | metformin and alogliptin | Kazano |
|  | metformin and canagliflozin | Invokamet |
|  | metformin and dapagliflozin | Xigduo |
|  | metformin and empagliflozin | Synjardy |
|  | metformin and linagliptin | Jentadueto |
|  | metformin and rosiglitazone | Avandamet |
|  | metformin and saxagliptin | Komboglyze |
|  | metformin and sitagliptin | Janumet, Velmetia |
|  | pioglitazone and alogliptin | Oseni, Incresync |
|  | saxagliptin and dapagliflozin | Qtern |

**Adapted from:**

Tu K, Manuel D, Lam K, Kavanagh D, Mitiku TF, Guo H. Diabetics can be identified in an electronic medical record using laboratory tests and prescriptions. *J Clin Epidemiol*. 2011 Apr; 64(4):431-5.

Ivers NM, Tu K, Young J, Francis JJ, Barnsley J, Shah BR, Upshur REG, Moineddin R, Grimshaw JM, Zwarenstein M. Feedback GAP: Pragmatic, cluster-randomized trial of goal setting and action plans to increase the effectiveness of audit and feedback interventions in primary care. *Implementation Sci*. 2013 Dec; 8:142.

**Appendix B:** Eligible OHIP Service Codes Classification

| **Visit Type** | **Billing Code** | **Description** |
| --- | --- | --- |
| In-Person Visit  (labelled as “Virtual Visit” if accompanied with the following Virtual Care Program service codes: B099, B100, B101, B102, B103, B200, B201, B202, B203) | A001 | minor assessment |
|  | A002 | enhanced 18-month well baby visit |
|  | A003 | major assessment |
|  | A004 | general re-assessment |
|  | A007 | intermediate assessment |
|  | A008 | mini assessment |
|  | A071 | complex medical specific re-assessment |
|  | A131 | complex medical specific re-assessment |
|  | A134 | medical specific re-assessment |
|  | A624 | medical specific re-assessment |
|  | A888 | partial assessment |
|  | A903 | pre-op assessment |
|  | A920 | medical management of early pregnancy, initial visit |
|  | K005 | primary mental health |
|  | K007 | ind. psychotherapy per half hour - gp |
|  | K013 | counselling-one or more people-per 1/2hr |
|  | K017 | periodic health visit-child aft. 2nd birthday |
|  | K022 | hiv prim care individ care 1/2 hr or major part |
|  | K028 | sexually transmitted disease (std) counseling |
|  | K030 | diabetic management fee |
|  | K032 | gp-specific neurocognitive assessment |
|  | K033 | counselling - 1 pt/yr/unit |
|  | K039 | smoking cessation follow-up visit |
| Virtual Visit | K080 | Minor assessment of patient by telephone or video |
|  | K081 | Intermediate assessment including psychotherapy by telephone or video |
|  | K082 | Psychotherapy, psychiatric or mental health counselling by telephone or video |
|  | K087 | Minor assessment of an uninsured by telephone or video |
|  | K088 | Intermediate assessment of an uninsured patient including psychotherapy by telephone or video |
|  | K089 | Psychotherapy, psychiatric or mental health counselling of an uninsured patient by telephone or video |

**Adapted from:**

Tu K, Sodhi S, Kidd M, et al. The University of Toronto Family Medicine Report: Caring for our Diverse Populations. 2020. Technical Appendix.

**Appendix C:** Repeated Cross-Sectional Design to identify pre-existing HbA1C level and BP for pre-pandemic, early pandemic and later pandemic time periods.

| Sep-18 | Oct-18 | Nov-18 | Dec-18 | Jan-19 | Feb-19 | Mar-19 | Apr-19 | May-19 | Jun-19 | Jul-19 | Aug-19 |
| --- | --- | --- | --- | --- | --- | --- | --- | --- | --- | --- | --- |
| Patient HbA1c levels and BP measurement | | | | | | Pre-pandemic outcome | | | | | |
|  |  |  |  |  |  |  |  |  |  |  |  |
| Sep-19 | Oct-19 | Nov-19 | Dec-19 | Jan-20 | Feb-20 | Mar-20 | Apr-20 | May-20 | Jun-20 | Jul-20 | Aug-20 |
| Patient A1c levels and BP measurement | | | | | | Early pandemic outcome | | | | | |
|  |  |  |  |  |  |  |  |  |  |  |  |
| Sep-20 | Oct-20 | Nov-20 | Dec-20 | Jan-21 | Feb-21 | Mar-21 | Apr-21 | May-21 | Jun-21 | Jul-21 | Aug-21 |
| Patient A1c levels and BP measurement | | | | | | Late pandemic outcome | | | | | |
